# Association between Myocardial Triglyceride Content and Cardiac Function in Healthy Subjects and Endurance Athletes

**DOI:** 10.1371/journal.pone.0061604

**Published:** 2013-04-16

**Authors:** Eiryu Sai, Kazunori Shimada, Takayuki Yokoyama, Shuji Sato, Tetsuro Miyazaki, Makoto Hiki, Yoshifumi Tamura, Shigeki Aoki, Hirotaka Watada, Ryuzo Kawamori, Hiroyuki Daida

**Affiliations:** 1 Department of Cardiology, Juntendo University Graduate School of Medicine, Tokyo, Japan; 2 Sportology Center, Juntendo University Graduate School of Medicine, Tokyo, Japan; 3 Department of Radiology, Juntendo University Hospital, Tokyo, Japan; 4 Department of Medicine, Metabolism and Endocrinology, Juntendo University School of Medicine, Tokyo, Japan; 5 Department of Radiology, Juntendo University Graduate School of Medicine, Tokyo, Japan; Virginia Commonwealth University, United States of America

## Abstract

Ectopic fat accumulation plays important roles in various metabolic disorders and cardiovascular diseases. Recent studies reported that myocardial triglyceride (TG) content measured by proton magnetic resonance spectroscopy (^1^H-MRS) is associated with aging, diabetes mellitus, and cardiac dysfunction. However, myocardial TG content in athletes has not yet been investigated. We performed ^1^H-MRS and cardiac magnetic resonance imaging in 10 male endurance athletes and 15 healthy male controls. Serum markers and other clinical parameters including arterial stiffness were measured. Cardiopulmonary exercise testing was also performed. There were no significant differences in clinical characteristics including age, anthropometric parameters, blood test results, or arterial stiffness between the two groups. Peak oxygen uptakes, end–diastolic volume (EDV), end–systolic volume (ESV), left ventricular (LV) mass, peak ejection rates and peak filling rates were significantly higher in the athlete group than in the control group (all P<0.02). Myocardial TG content was significantly lower in the athlete group than in the control group (0.60±0.20 vs. 0.89±0.41%, P<0.05). Myocardial TG content was negatively correlated with EDV (*r* = −0.47), ESV (*r* = −0.64), LV mass (*r* = −0.44), and epicardial fat volume (*r* = 0.47) (all P<0.05). In conclusion, lower levels of myocardial TG content were observed in endurance athletes and were associated with morphological changes related to physiological LV alteration in athletes, suggesting that metabolic imaging for measurement of myocardial TG content by ^1^H-MRS may be a useful technique for noninvasively assessing the “athlete’s heart”.

## Introduction

Ectopic fat accumulation is associated with various metabolic disorders and cardiovascular diseases [1]–[3]. Previous animal studies have shown that myocardial triglyceride (TG) accumulation triggers pathological changes, including myocardial apoptosis and ventricular systolic dysfunction [4], [5]. However, the assessment of myocardial TG content is hampered by the difficulty of obtaining myocardial tissues in a clinical setting.

Recent studies have demonstrated that proton magnetic resonance spectroscopy (^1^H-MRS) enables the noninvasive monitoring of TG accumulation in human myocardial tissue. Indeed, myocardial TG content, as measured by ^1^H-MRS, has been associated with aging [6], diabetes mellitus [7], myocardial systolic dysfunction [4], [8], [9], and diastolic dysfunction [6], [10]. In addition, caloric restriction induced a dose-dependent increase in myocardial TG content [11], whereas endurance training reduced myocardial TG content [12]. However, myocardial TG content in endurance athletes has not yet been investigated.

The purpose of this study is to evaluate the associations between myocardial TG content, cardiac morphology and left ventricular (LV) function assessed by ^1^H-MRS and magnetic resonance imaging (MRI) in healthy subjects and endurance athletes.

## Methods

### Subjects

Fifteen healthy male subjects and 10 male endurance athletes were recruited by advertisements in a local area. All subjects were non-obese, aged 20–40 years, and without acute or chronic disease. Subjects receiving medical treatment, current smokers, and those with abnormal laboratory parameters were excluded. We defined an endurance athlete as a person who performed endurance training for more than 5 days a week, and was affiliated with a specific athletic association to participate in competitive sports such as cycling, track, or swimming. The international physical activity questionnaire (IPAQ) was used to assess each subject’s activity level [13]. All protocols were approved by the ethical committee of the Juntendo University, and all participants provided written informed consent before their participation in this study according to the guidelines established in the Declaration of Helsinki.

### Measurements of Body Composition

Skeletal muscle mass and body fat weight were measured after overnight fasting by multi-frequency bioelectrical impedance analysis using eight tactile electrodes (MF-BIA8; In-Body 720, Biospace, Korea) [14] after overnight fasting. The subject stood on the footplate with barefoot and held the electrodes in both hands. This process takes 2 min, and measurement requires no specific skills. The apparatus then automatically displays measurements of fat-free mass, fat mass, and percentage body fat.

### Blood Measurements

Standard laboratory tests including blood cell counts, fasting plasma glucose, lipids, creatinine, free fatty acid, and N-terminal pro-brain natriuretic peptide (NT-proBNP) were performed immediately before MRS after overnight fasting. Serum lipid profiles were measured using specific assays for total cholesterol (Symex Co, Kobe, Japan), triglyceride (Sekisui Medical, Tokyo, Japan), and high-density lipoprotein cholsterol (Sekisui Medical, Tokyo, Japan) by BioMajesty JCA-BM8060 analyzer (Japan Electron Optics Laboratory Ltd, Tokyo, Japan). Serum low-density lipoprotein cholesterol levels were calculated using the Friedewald’s formula. Serum insulin was measured by chemiluminescent enzyme immunoassay using the Lumipulse presto II analyzer (Fujirebio Inc, Tokyo, Japan). A homeostasis model assessment index (HOMA-IR) was calculated to estimate insulin resistance from fasting insulin and glucose concentrations: insulin (µU/ml)×glucose (mmol/l)/22.5. Free fatty acid (FFA) was measured a standard enzymatic assay (Eiken chemical Co. Ltd, Tokyo, Japan) by BioMajesty JCA-BM2250 analyzer (Japan Electron Optics Laboratory Ltd, Tokyo, Japan). Serum NT-proBNP was determined using an electrostatic controlled linear inchworm actuator on Hitachi modular analytics (HITACHI Hi-Technologies Co. Ltd. Tokyo, Japan). HbA1c concentrations were measured in whole blood samples using latex-enhanced immunoassay (Fujirebio Co. Ltd. Tokyo, Japan).

### MRI and MRS

All cardiac MRI and ^1^H-MRS studies were performed using a MAGNETOM Avanto 1.5-Tesla MRI system (Siemens Medical Solution, Erlangen, Germany) with subjects resting in the supine position. To minimize the influence of breathing, a towel was strapped around the subject’s upper abdomen. Dynamic cine images were used to determine LV mass, and LV functional parameters. Image analysis was performed using special evaluation software (Argus; Siemens Medical Systems, Erlangen, Germany) [15], [16] on a separate work station. Endocardial and epicardial LV borders were traced manually at end-diastole and end-systole from short-axis cine images. End-diastolic volume (EDV), end-systolic volume (ESV), stroke volume, and ejection fraction were calculated by Simpson’s method. In addition, the peak LV ejection and filling rates were automatically derived on the basis of LV volume-time curves. The area of epicardial fat was traced on consecutive end diastolic short axis images, beginning with the most basal slice at the level of the mitral valve, and moving apically through the stack until the most inferior margin of adipose tissue, as reported previously [17].

**Figure 1:**
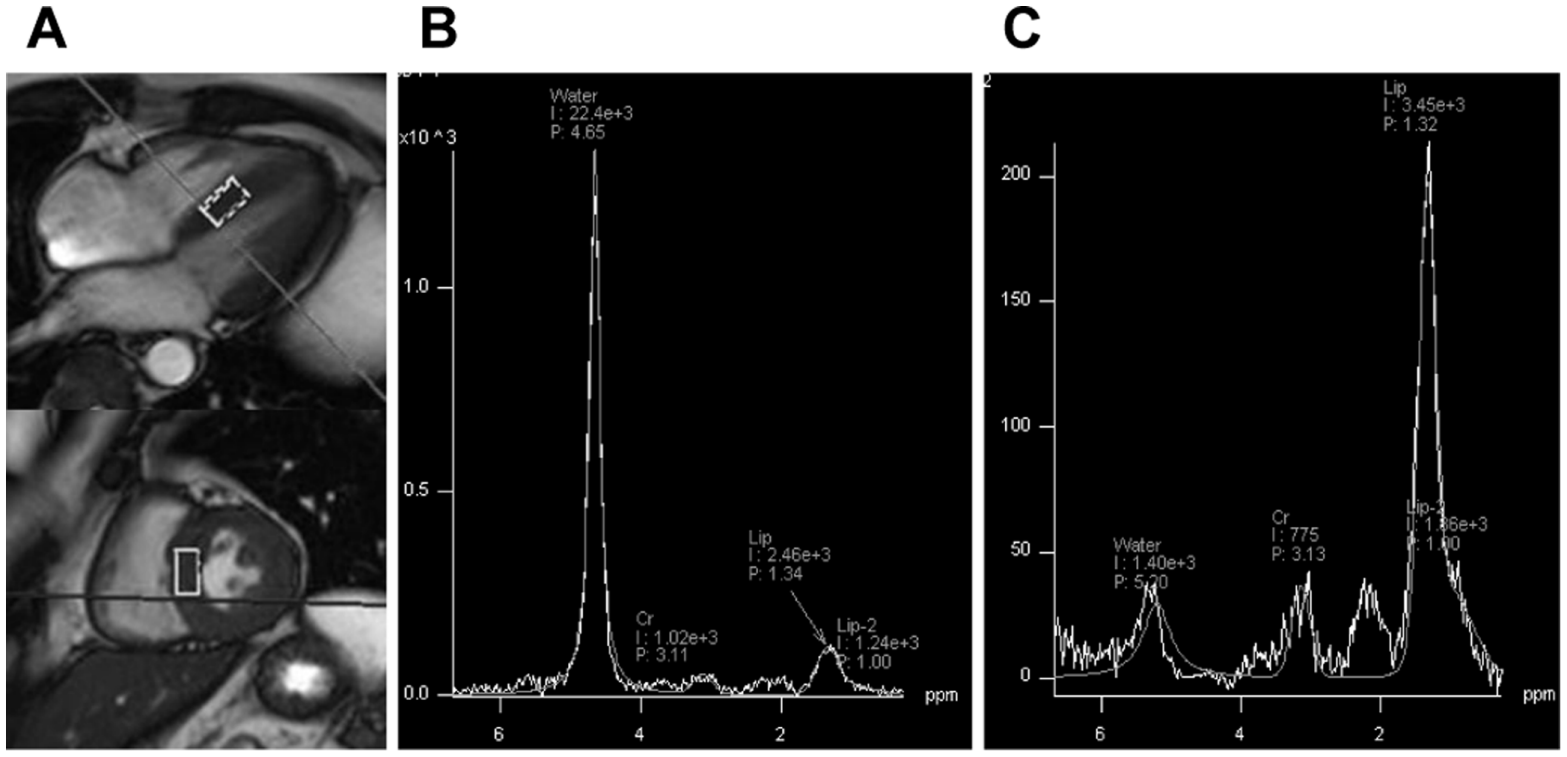
Representative results of H^1^-MR spectra in a healthy subject. A: Myocardial voxel localization for H^1^-MRS in 4-chamber and short axis views. B: H^1^-MR spectra without water suppression. C: H^1^-MR spectra without water suppression.

After the cine MRI imaging, myocardial TG content was determined by ^1^H-MRS. A volume of interest (VOI = 2.0 cm^3^−10×10×20 mm) was selected within the ventricular septum from cine dynamic cine-mode images of the heart (Figure 1). We adjusted the VOI size to the anatomy of the ventricular septum. The spectrum of water and lipid was acquired by point-resolved spectroscopy (PRESS) method using an echo time (TE) of 30 ms, and repetition time (TR) of at least 4,000 ms, myocardial TG signals were acquired at 1.4 ppm from spectra with water suppression, and water signals were acquired at 4.7 ppm from spectra without water suppression (Figure 1). Areas under the curves for water and lipid peaks were quantified using standard line-fitting procedures (Siemens Syngo Spectroscopy). Myocardial TG level was expressed as a ratio of lipid to water (%). Thus, ^1^H-MRS evaluation of myocardial TG content was performed essentially as has been previously validated [18]–[21].

### Measurement of Cardiopulmonary Fitness

All subjects underwent an incremental cycling test (Corival 400, Lobe B.V., Groningen, Netherlands) using an expiratory gas analyzer (Vmax-295, sensorMedics Co., Yorba Linda, CA, USA) to measure anabolic threshold (AT) and maximal oxygen consumption (VO_2max_). After a 3-min rest period, a warm-up was performed for 3 minutes at 40 W, followed by ramp loading (15–30 W/min) until the subjective exhaustion, as described previously [22]. According to the ATS/ACCP guidelines, AT was determined by V-slope method. In cases when AT was not identified on the V-slope, we used the point at which V_E_/VO_2_ starts to increase while V_E_/VCO_2_ remains constant [23].

### Evaluation of Atherosclerotic Parameters

The cardio ankle vascular index (CAVI) was measured as atherosclerotic parameters. CAVI was automatically calculated by VaSera VS-1500AN (Fukuda Denshi Co. Ltd., Tokyo, Japan) [24], [25].

### Statistical Analyses

Values are expressed as mean ± standard deviation (SD). For variables that did not show a normal distribution, the data were transformed into natural logarithmic values before statistical analyses. Correlations were calculated using Pearson’s correlation co-efficient. Unpaired Student’s *t*-test was used to compare groups. All statistical analyses were performed with SPSS version 20 (SPSS, Inc). A P value of less than 0.05 was considered significant.

## Results

The clinical characteristics of study subjects are summarized in Table 1. There were no significant differences, in age, body composition, lipids, glucose, insulin levels, or NT-proBNP between the two groups. The levels of AT (29.2±6.6 ml/kg/min vs. 19.0±5.2 ml/kg/min, P* = *0.0002), VO_2max_ (52.3±6.2 ml/kg/min vs. 43.2±8.0 ml/kg/min, P = 0.0057) and international physical activity questionnaire (IPAQ) score (2318±1605 vs. 5310±2869, P = 0.0048) were significantly higher in the athlete groups than in the control group.

**Table 1:** Clinical Characteristics.

	Control group(n = 15)	Athlete group(n = 10)	P value
Age, years	28.8±4.5	26.4±4.4	0.20
Body height, m	1.735±0.051	1.732±0.047	0.88
Body weight, kg	67.9±7.4	67.8±4.2	0.94
Body mass index, kg/m^2^	22.5±1.9	22.6±1.9	0.90
Skeletal muscle mass, kg	30.7±2.6	32.5±2.0	0.083
Body fat weight, kg	13.6±3.8	10.6±3.6	0.066
Percent of body fat, %	18.6±5.0	15.4±4.8	0.14
Neck circumference, cm	36.9±2.4	36.8±1.8	0.92
Waist circumference, cm	80.5±6.8	78.1±4.0	0.36
Total cholesterol, mg/dl	174.6±26.3	182.5±24.5	0.45
Triglyceride, mg/dl	74.6±27.0	61.1±15.8	0.16
LDL-cholesterol, mg/dl	104.2±26.4	111.1±29.0	0.53
HDL-cholesterol, mg/dl	55.7±11.3	59.2±12.7	0.47
Fasting free fatty acid, µEq/L	299.1±132.3	364.7±211.5	0.32
Fasting blood glucose, mg/dl	90.7±8.6	90.9±5.0	0.93
Insulin, µU/ml	5.6±3.0	4.4±1.4	0.22
HOMA-IR	1.3±0.6	1.0±0.3	0.22
HbA1c, %	4.7±0.3	4.7±0.2	0.51
Creatinine, mg/dl	0.84±0.10	0.84±0.05	0.85
eGFR, ml/min/m^2^	91.6±12.2	92.1±6.7	0.91
NT-proBNP, ng/l	18.6±18.0	10.1±3.9	0.15
Urinary acid, mg/l	6.0±0.9	5.4±1.3	0.15
Anaerobic threshold,ml/kg/min	19.0±5.2	29.2±6.6	0.0002
VO_2_max, ml/kg/min	43.2±8.0	52.3±6.2	0.0057
CAVI	6.5±0.7	6.2±0.6	0.53
IPAQ score	2318±1605	5310±2869	0.0048

Values are mean ± SD. bpm = beats per minutes, LDL = low-density lipoprotein; HDL = high-density lipoprotein; eGFR = estimated glomerular filtration rate; HOMA-IR = homeostasis model assessment of insulin resistance, NT-proBNP = N-terminal pro brain natriuretic peptides, VO_2_max = maximal oxygen intake, CAVI = cardio ankle vascular index, IPAQ = international physical activity questionnaire.

P value denotes significance of unpaired *t* test between athlete group and healthy control.

**Figure 2:**
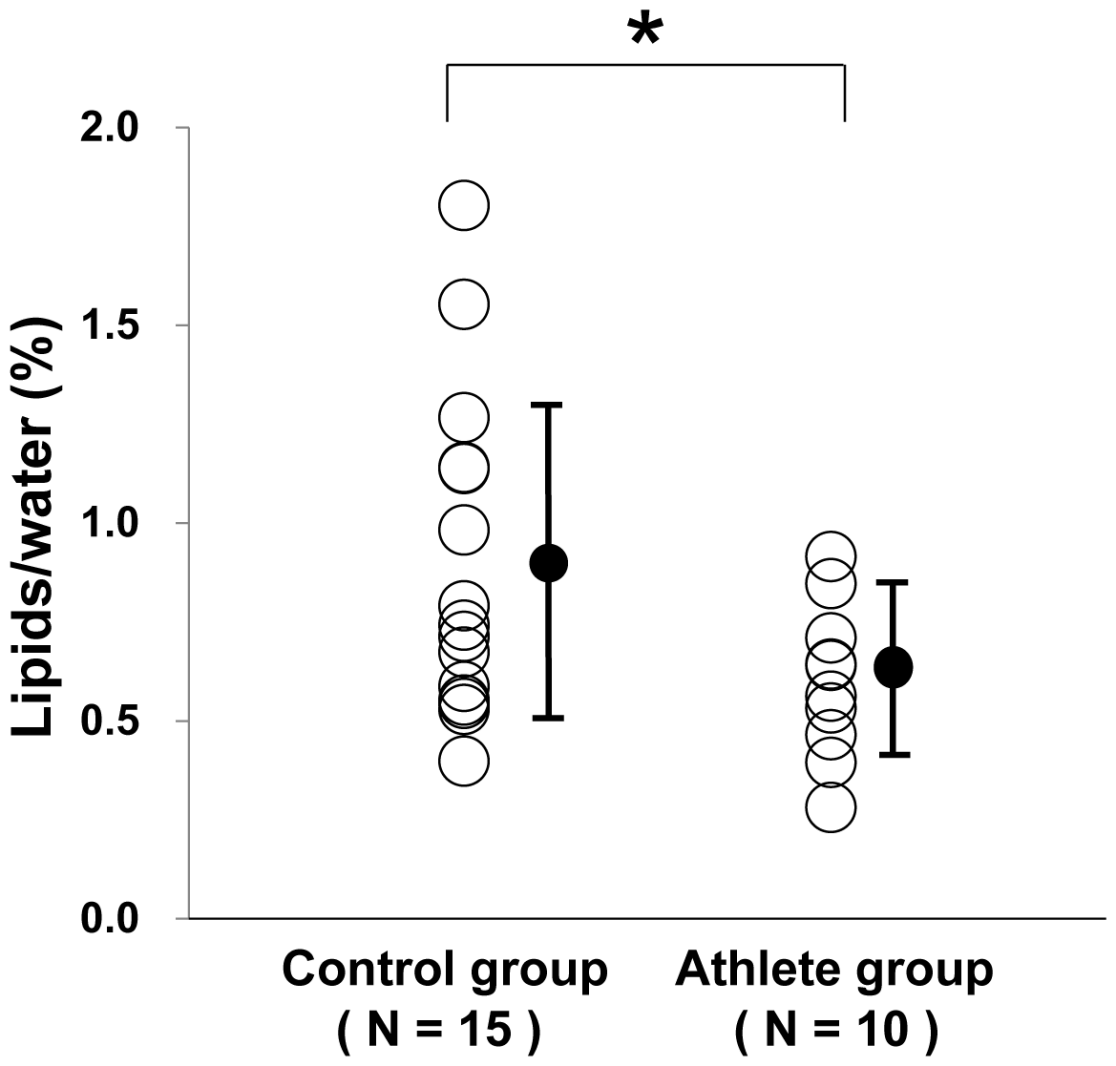
Comparison between myocardial TG content in the control group and the athlete group. * *P*<0.05 between the two groups.

MRI and MRS variables are shown in Table 2. The values of EDV (182±24 ml vs. 153±16 ml, P = 0.0011), ESV (96±16 ml vs. 73±8 ml, P = 0.0002), and LV mass (139±16 g vs. 120±13 g, P = 0.0034), were significantly higher in the athlete group than in the control group. Peak ejection rate (777±230 ml/sec vs. 551±206 ml/sec, P = 0.019) and peak filling rate (839±250 ml/sec vs. 619±177 ml/sec, P = 0.018) were significantly higher in the athlete group than in the control group. None of the subjects had an abnormal peak ejection or filling rate. Myocardial TG content was significantly lower in the athlete group than in the control group (0.60±0.20% vs. 0.89±0.41%, P = 0.045) (Figure 2).

**Table 2:** MRI variables.

	Control group(n = 15)	Athlete group(n = 10)	P value
LV ejection fraction, %	50.6±5.5	48.1±6.3	0.32
LV end diastolic volume, ml	153±16	182±24	0.0011
LV end systolic volume, ml	73±8	95±16	0.0002
Stroke volume, ml	80±14	88±17	0.22
Cardiac output	4.8±0.8	5.2±1.2	0.29
LV myocardial mass, g	120±13	139±16	0.0034
Peak ejection rate, ml/sec	551±206	777±230	0.019
Peak filling rate, ml/sec	619±177	839±250	0.018
Epicardial fat volume, ml	48.8±14.8	38.3±8.2	0.057

Values are mean ± SD. LV = left ventricular.

P value denotes significance of unpaired *t* test between athlete group and healthy control.

**Figure 3:**
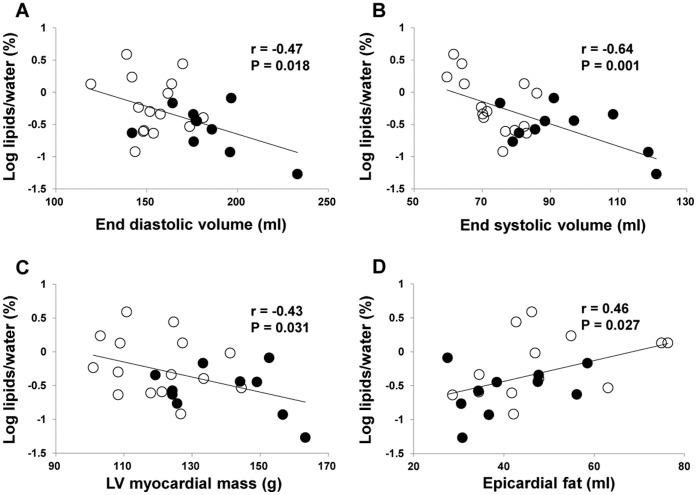
Correlations between myocardial TG content and MRI parameters. A: A correlation between myocardial TG content and end-diastolic volume. B: A correlation between myocardial TG content and end-systolic volume. C: Correlation between myocardial TG content and left ventricular (LV) mass. D: Correlation between myocardial TG content and epicardial fat volume. Open circle; control group. Closed circle; athlete group.

Myocardial TG content was negatively correlated with EDV (*r* = −0.47, P = 0.018), ESV (*r* = −0.64, P = 0.001), LV mass volume (*r* = −0.43, P = 0.031), and epicardial fat volume (*r* = 0.47, P = 0.025) (Figure 3). Although a significant correlation between myocardial TG content and VO_2max_ was not found (*r* = −0.15, P = 0.46), epicardial fat volume was negatively correlated with EDV, a LV morphological parameter (*r* = −0.44, P = 0.022).

## Discussion

The present study demonstrated that myocardial TG content was significantly lower in the endurance athlete group than in the control group and that myocardial TG content was significantly correlated with EDV, ESV, LV mass, and epicardial fat volume. This study is, to the best our knowledge, the first report to demonstrate an association between TG content and physiological LV alteration in endurance athletes.

Much attention has been focused on the associations between ectopic fat accumulation, various metabolic disorders and cardiovascular diseases [1], [2]. It has been reported that the myocardial TG content is associated with metabolic disorders [7], [26]. The positive correlation between myocardial TG content and LV mass has also been reported among the diabetic patients as well as in obese individuals with insulin resistance [4], [8]. Animal studies have demonstrated that myocardial TG content was associated with not only cardiovascular risk factors, but also with lipotoxicity-induced heart failure and premature death [20], [27]. In addition, increased myocardial TG content induced pathological LV hypertrophy, cardiac dysfunction, and non-ischemic dilated cardiomyopathy [28]. However, the present study showed negative correlations between myocardial TG and LV mass as well as LV function. Several studies suggested that mitochondrial dysfunction in the myocardium exists in patients with diabetes and insulin resistance [29]. In contrast, the functional capacity of mitochondria in athlete’s heart was reported to be increased by endurance training [30]. This difference in mitochondrial function may underlie the difference in myocardial TG content between the physiological modifications present in athlete’s heart and the pathological changes that characterize the deteriorating heart in patients with diabetes and insulin resistance.

Previous studies reported the relationship between exercise and lipid content in skeletal muscle. High levels of intra-myocellular lipid (IMCL) were reported in the skeletal muscles of patients with diabetes mellitus [31] and elderly subjects [32]. On the other hand, it has also been reported that similar high levels of IMCL occur in skeletal muscles of athletes, despite the marked insulin sensitivity and the high oxidative capacity of these muscles, this is the so-called “athlete’s paradox” [33]. Increases in IMCL content provide a substrate for energy metabolism during exercise [34]. A high availability of fatty acids is needed to augment TG resynthesis in skeletal muscle during and after exercise [34]. Diacylglycerol and/or ceramide, but not TG, may be directly associated with the development of insulin resistance [35], [36]. In the present study, no “athlete’s paradox” was observed in the subjects’ cardiac muscles. Several potential reasons have been raised. One possibility is the difference in mitochondrial function with regard to fatty acid metabolism between skeletal muscle and cardiac muscle. Fatty acid metabolism may be more efficient in cardiac muscles, which has more abundant mitochondria than in skeletal muscles [37]. Another reason relates to the differences in regulation of fatty acid β-oxidation between the two types of muscle. To sustain contractile function in the heart requires a greater energy supply [38]. Therefore, the fatty acid β-oxidation system in cardiac muscle is very dynamic and sufficient to meet the energy demands of the heart. Alterations in lipoprotein lipase (LPL) synthesis as well as the activation, secretion, transportation, capillary luminal binding, and the degradation of fats in cardiac myocytes, contribute to myocardial fatty acid supply, uptake and fatty acid β-oxidation [38]. In addition, the heart muscle is reported to be less susceptible to developing insulin resistance than skeletal muscle [39]. Therefore, insulin responsiveness and its consequences in the heart may be relatively high in endurance athletes.

A recent study has shown that acute endurance exercise leads to increased myocardial TG content depending on elevated plasma free fatty acid concentrations and the uptake of free acids in the heart. The mechanism is considered to be related to the increased availability of fatty acid during exercise in fasting healthy males [40]. The level of circulating free fatty acids concentration was low in the present study. Thus, fatty acid availability must be relatively low in these individuals. Indeed, myocardial TG content was not reported to change even after exercise in subjects with a suppressed state of free fatty acid synthesis [40]. In addition, endurance training regulates the activity of LPL [41], which provides the major source of free fatty acids derived from TG content lipoproteins. Endurance athletes manifesting physiological LV adaptations may be augmented to drive alterations in fatty acid metabolism on fasting state.

We measured several TG-associated enzymes and proteins, including adiponectin, pre-heparin LPL, apolipoprotein (apo) CII, and apo CIII. No significant difference was observed between the two groups for each parameter (data not shown). One of the major reasons, why these enzyme and proteins were not significantly different, is supposed to the study subjects consisting with healthy lean young men without any metabolic disorder. Myocardial lipid metabolism is regulated by a complex balance between fatty acid supply to the heart, competing energy substrates, energy demand and oxygen supply to the heart, uptake and esterification of fatty acid, and control of mitochondrial functions such as fatty acid oxidation and electron transport chain activity [38]. In addition, epicardial fat, which stores free fatty acid during excessive circulating free fatty acid accumulation and releases fatty acid when energy is needed, is directly connected to the myocardium. Accordingly, we report a significant positive correlation between epicardial fat volume and myocardial TG content. It has reported that the metabolic rates of lipolysis and lipogenesis are 2-fold higher in epicardial fat than in other fat deposits. Indeed, we detected a negative correlation between epicardial fat volume and EDV, a LV morphology parameter. The precise mechanism underlying the low myocardial TG content in endurance athletes remains elusive. However, the significant positive correlation between epicardial fat volume and myocardial TG content may be related to the increase of utilizing fatty acid in endurance athletes. In our next step, we plan to clarify the impact of exercise on myocardial TG content and LV alterations in endurance athletes.

### Limitations

The present study has several limitations. First, this was a single center study with a small sample size, studies of larger sample size are required to confirm these findings. Second, this study included only male subjects. Third, a previous study has demonstrated that a negative relationship between myocardial TG content and cardiopulmonary fitness in obese women [26]. This correlation between myocardial TG content and VO_2max_ was not found in our study. This discrepancy may have resulted from the difference between the subjects in these studies, as in the present study, all subjects of the present study were healthy males without metabolic disorders. Finally, athlete’s heart is considered to be reversible [42], therefore, we will next evaluate the effect of detraining on myocardial TG content.

### Conclusions

Low levels of myocardial TG content were observed in endurance athletes and were associated with the morphology of physiological LV alteration. These data suggest that metabolic imaging for measurement of myocardial TG content by ^1^H-MRS may be a useful technique for noninvasively assessing the “athlete’s heart”.
